# Content Validation of a Bilingual Screening Tool for Identifying Stage-Specific Cervical Spondylosis Symptoms and Work Patterns Among Computer Professionals

**DOI:** 10.7759/cureus.105509

**Published:** 2026-03-19

**Authors:** Anupam Garg, Jasmine Anandabai

**Affiliations:** 1 Physiotherapy, Jyotirao Phule Subharti College of Physiotherapy, Swami Vivekanand Subharti University, Meerut, IND; 2 Faculty of Allied Health Sciences, Swami Vivekanand Subharti University, Meerut, IND

**Keywords:** bilingual screening tool, cervical spondylosis, computer professionals, content validity, musculoskeletal symptoms, occupational health, work patterns

## Abstract

Background: Computer professionals are at increased risk of cervical spine disorders due to prolonged static postures, repetitive movements, and occupational exposure. Early identification of stage-specific symptoms and associated work patterns is important for preventive physiotherapy and occupational health planning. A bilingual (English-Hindi) self-reported screening tool was developed to identify eligible participants, detect stage-specific symptoms of cervical spondylosis, and identify work patterns.

Objective: To establish the expert content validity of a bilingual screening tool designed to identify stage-specific cervical spondylosis symptoms and work patterns among computer professionals.

Methodology: A descriptive content validation study was conducted using 11 clinical and academic experts. The tool followed a progression-based structure to identify Stage 1 (cervical degenerative disc disease (CDDD)) and Stage 2 (cervical spondylotic radiculopathy (CSR)) symptoms, with work patterns recorded as sedentary, mobile, and hybrid. Content validity was assessed using Item-Content Validity Index (I-CVI) and Scale-Content Validity Index (S-CVI/Ave), with an acceptable threshold of ≥0.78 for six or more experts. Modified kappa statistics were calculated to account for chance agreement.

Results: All items demonstrated high expert agreement. I-CVI values ranged from 0.91 to 1.00, and S-CVI/Ave was 0.99, indicating acceptable CVIs according to established benchmarks. Modified kappa values indicated agreement beyond chance. Qualitative feedback indicated clarity and perceived usability of the instrument for self-reported screening.

Conclusions: The bilingual screening tool demonstrated high expert agreement and acceptable CVIs for identifying stage-specific symptoms and work patterns among computer professionals. Its structured progression-based design may support occupational screening and practical application in workplace and community settings.

## Introduction

Cervical spondylosis is increasingly observed among computer professionals, where prolonged static postures, repetitive neck movements, and sustained screen exposure act as significant occupational stressors [1]. Occupational exposure, workstation posture, and repetitive tasks significantly contribute to neck pain and related symptoms among office-based workers [2]. The risk and clinical manifestations of musculoskeletal disorders vary across professions because each occupation involves distinct physical demands and exposure patterns [3].

Various clinical classifications for cervical spondylosis exist; however, they often lack uniformity and structured staging suitable for early screening and symptom identification [4]. Most available tools focus primarily on disability assessment rather than structured stage-specific symptom recognition.

Early identification of degenerative and radicular symptoms is essential because conservative physiotherapy and ergonomic correction are most effective in the initial stages [5]. Advanced clinical presentations, particularly Stage 3 (Cervical Spondylotic Myelopathy), were excluded from screening as contemporary clinical practice guidelines recommend specialized surgical evaluation rather than community-level screening for progressive myelopathy [6].

The progression of cervical spondylosis symptoms generally moves from localized discomfort to radicular involvement, reflecting the natural disease course [7]. Self-reported screening instruments, when properly adapted to local language and context, improve accuracy and participation in large population studies [8].

Existing clinical instruments, such as the Neck Disability Index (NDI), primarily quantify the severity of disability in symptomatic individuals. In contrast, the bilingual screening instrument developed in the present study is intended to identify stage-specific symptom patterns and their association with occupational work patterns among computer professionals. This approach facilitates early risk identification and structured clinical screening before substantial functional disability develops.

This study addresses a critical gap by validating a bilingual self-reported screening tool designed to identify Stage 1 (Cervical Degenerative Disc Disease (CDDD)) and Stage 2 (Cervical Spondylotic Radiculopathy (CSR)) symptoms, along with structured work pattern categorization among computer professionals. The tool follows a progression-based structure and provides a systematic framework for screening and occupational health research. The clinical staging and work pattern categories were operationally defined for this instrument based on a focused literature synthesis and clinical reasoning, with the intent of enabling structured occupational screening.

## Materials and methods

This descriptive content validation study was conducted to evaluate the clarity, relevance, and applicability of a bilingual (English-Hindi) screening tool developed for computer professionals.

The instrument was designed to screen participants based on inclusion and exclusion criteria, identify stage-specific symptoms of cervical spondylosis, and capture work patterns categorized as sedentary, mobile, or hybrid.

The tool focused on Stage 1 (CDDD) and Stage 2 (CSR). Stage 3 (Cervical Myelopathy, CM) was excluded, as such cases commonly require surgical evaluation and fall outside the scope of preventive physiotherapy screening.

Symptoms were organized in a staged format with Stage 1 symptoms presented first, followed by Stage 2 symptoms arranged in a clinically logical sequence to support self-identification.

Items were developed through the synthesis of existing literature on cervical spondylosis and occupational neck pain among computer professionals, followed by clinical refinement. The initial draft was internally reviewed for wording, bilingual clarity, and logical sequencing before expert content validation. The structural overview of the validated bilingual screening tool is provided in Appendix.

The validation panel comprised 11 physiotherapy subject experts with diverse academic and clinical experience across musculoskeletal, orthopedic, neurological, and rehabilitation physiotherapy, including cervical spine-related conditions. These experts were selected according to predefined criteria, including postgraduate or doctoral qualifications and a minimum of five years of clinical or academic experience in musculoskeletal physiotherapy, particularly cervical spine disorders. Following established methodological recommendations for panels consisting of six or more experts, an item-level content validity index (I-CVI) threshold of 0.78 was used to determine acceptable agreement among experts [9,10]. Most experts held postgraduate or doctoral qualifications and were actively involved in academic teaching, research, and clinical practice. The panel size exceeded the recommended minimum requirements for content validation studies [9]. Experts independently evaluated each item for relevance, clarity, simplicity, and appropriateness, and endorsements indicating item acceptability were used to compute the CVI.

Content validity was assessed using descriptive validation statistics. No hypothesis testing was performed. The content validation process followed established recommendations for expert content validity procedures and CVI reporting [10]. I-CVI and scale-content validity index (S-CVI/Ave) were calculated, where I-CVI represents the proportion of experts endorsing an item as acceptable and S-CVI/Ave represents the average of I-CVI values across items. Following established methodological recommendations for content validation panels comprising six or more experts, an I-CVI value of 0.78 or higher was considered acceptable, consistent with classical and contemporary content validity literature [9,10].

To account for chance agreement, modified kappa (k*) was calculated using item-wise agreement counts and established content validity agreement formulas; k* ranged from approximately 0.91 to 1.00.

Ethical approval was obtained from the Institutional Ethics Committee.

**Figure 1:**
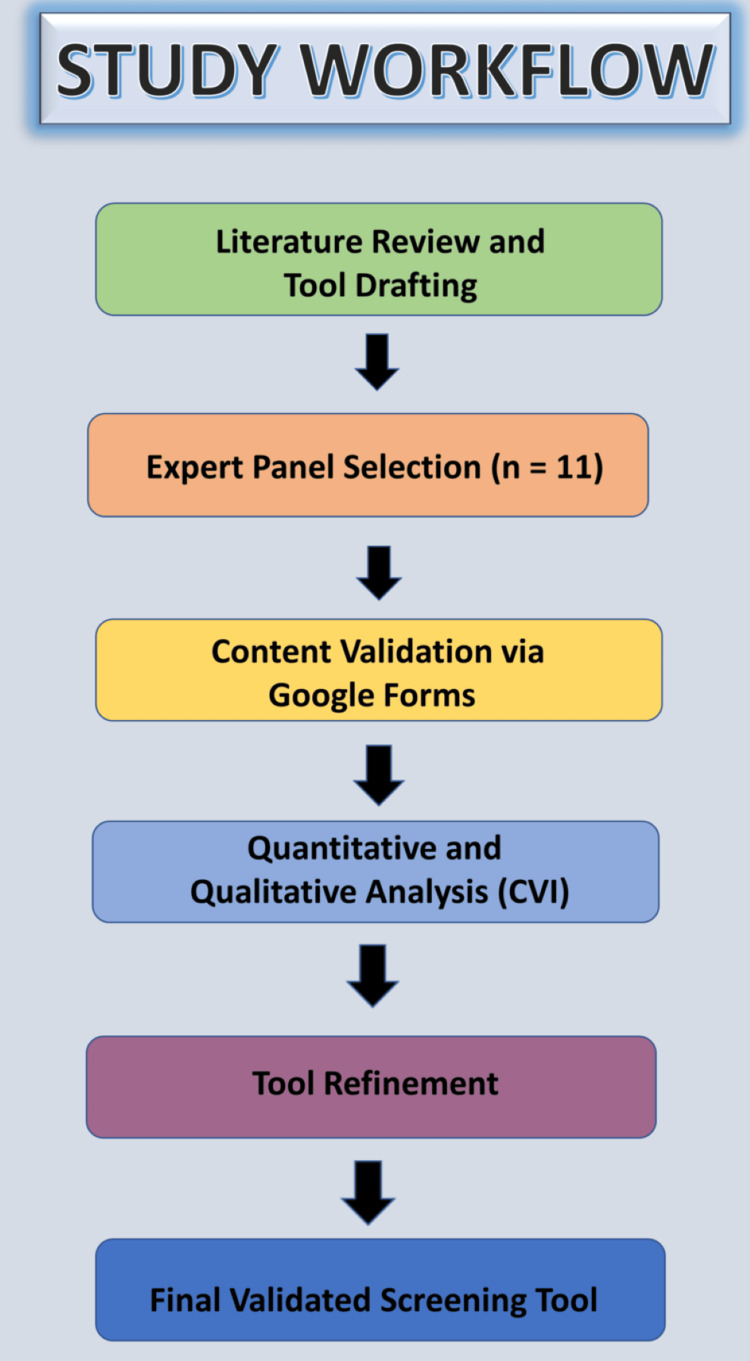
Study workflow for the development and content validation of the bilingual screening tool. The figure outlines the sequential steps, including literature review, tool drafting, expert panel selection, content validation using a structured online questionnaire, quantitative and qualitative analyses, and final refinement of the screening tool. Image credit: All authors.

Experts were invited via email with study details and the preliminary bilingual screening tool attached as a PDF. Expert responses were collected using a structured online form (Google Forms) incorporating an expert declaration statement. Submission of the completed form was considered as implied informed consent for participation. Qualitative feedback was summarized descriptively (Figure 1).

## Results

All items demonstrated high expert agreement. The I-CVI values ranged from 0.91 to 1.00, indicating strong relevance and clarity of the instrument items. The overall S-CVI/Ave score was 0.99, reflecting excellent scale-level content validity. Modified kappa coefficients demonstrated excellent agreement beyond chance, ranging from 0.91 to 1.00.

Nine items achieved an I-CVI of 1.00, while the item related to terminology demonstrated an I-CVI of 0.91, which remained within acceptable validity thresholds.

Qualitative expert feedback indicated that the tool was easy to understand, clinically relevant, and suitable for self-reported screening in occupational settings. The bilingual structure improved accessibility and comprehension, particularly for non-medical computer professionals.

Detailed quantitative findings are presented in Table 1, while qualitative expert feedback was summarized descriptively and is presented in Table 2.

**Table 1:** Quantitative content validity index (CVI) of the bilingual screening tool (n = 11).

Validation item	Agree	Disagree	I-CVI
Structure and sequence	11	0	1.00
Work pattern relevance	11	0	1.00
Inclusion and exclusion criteria	11	0	1.00
Stage-wise symptoms	11	0	1.00
Symptom list completeness	11	0	1.00
Severity scale (0–3)	11	0	1.00
Bilingual format clarity	11	0	1.00
Medical versus layman terminology	10	1	0.91
Digital and offline usability	11	0	1.00
Overall validity and fitness for use	11	0	1.00

**Table 2:** Summary of qualitative expert feedback and implementation. Implemented = Suggestion incorporated into the final version of the screening tool. Not implemented = Suggestion reviewed but not included to preserve consistency and avoid response bias.

Expert suggestion	Action taken	Scientific justification
Reduction of medical terminology	Implemented	Layman-friendly explanations were added alongside standard medical terms to enhance participant comprehension and improve the accuracy of self-reported responses without compromising analytical consistency.
Improvement of Hindi language clarity	Implemented	Hindi wording was refined to ensure grammatical accuracy and uniform comprehension across participants with varied educational backgrounds.
Re-structuring of the *Basic Details* section	Implemented	Demographic details and work-profile information were separated to improve logical flow, data organization, and ease of correlation during analysis.
Clarification of symptom descriptions	Implemented	Symptom explanations were expanded to reduce ambiguity while retaining the original clinical meaning and stage-wise intent.
Inclusion of visual aids (e.g., dermatome/myotome maps)	Not implemented	The instrument is a self-report screening tool; inclusion of anatomical images may increase interpretation variability and introduce response bias among non-clinical participants.
Overall simplification of the tool layout	Implemented	Formatting and layout adjustments were made to enhance readability and usability without altering item content, scoring, or construct validity.

Minor wording and organizational refinements were additionally performed by the authors to enhance clarity; these did not alter item content or scoring and therefore did not require re-validation.

## Discussion

The excellent validity indices obtained in this study support the utility of the tool for structured screening among computer professionals. The progression-based arrangement of symptoms reflects the natural course of cervical spondylosis and facilitates identification of early degenerative and radicular symptoms through self-reporting. The high I-CVI and S-CVI/Ave values indicate excellent item relevance and scale-level content validity according to established benchmarks. The high modified kappa values further confirm strong expert agreement beyond chance.

By intentionally excluding Stage 3 (Cervical Myelopathy), the instrument supports focused identification of early-stage symptom patterns, where preventive physiotherapy and ergonomic interventions are most effective.

In the present study, the bilingual design improved usability across diverse populations and enhanced participation in occupational health screening programs. Cross-cultural adaptation has been shown to improve accuracy in self-reported health instruments [8].

Another important strength is the integration of work pattern identification. Since occupational exposure varies significantly across professions and work types, structured identification supports exploration of symptom distribution across professional activity patterns [2]. The work pattern framework was developed based on existing literature and occupational health considerations relevant to computer professionals.

The findings of this study demonstrate high expert-based content validity for the bilingual screening instrument. However, these findings represent an early phase of instrument development. Although the tool shows potential for identifying stage-specific cervical spondylosis symptoms and associated occupational risk factors among computer professionals, further empirical testing and field validation in larger and more diverse populations will be necessary before it can be adopted for routine clinical or occupational screening.

Limitations

Limitations of this study include the absence of construct validation, reliability testing, and field-based pilot testing in the target population, as the present work was limited to expert content validation. The expert panel approach may introduce potential subjective bias related to professional background and interpretation of item wording. Responsiveness and criterion-related validity were not assessed. Future studies should evaluate internal consistency, test-retest reliability, construct validity, and feasibility in workplace screening settings.

## Conclusions

This study demonstrates satisfactory expert-based content validity for a bilingual screening tool designed to identify stage-specific cervical spondylosis symptoms and associated work patterns among computer professionals. The instrument may support structured preliminary screening in occupational and community settings. Further psychometric evaluation, including reliability and construct validation, is recommended before broader implementation.

## Appendices

Appendix 

**Table 3:** Structural Overview of the Validated Bilingual Screening Tool This appendix presents only the section-wise structure of the validated bilingual screening tool to provide an overview of its organization and domains. The final validated screening tool is available with the authors and can be accessed upon reasonable academic request.

Section	Title	Purpose & Content
Section A	Demographic Details	Captures age group, gender, and residential area.
Section B	Work Profile	Assesses computer-based job status, experience duration, and daily working hours.
Section C	Diagnosis History	Records any prior clinical diagnosis of cervical spondylosis.
Section D	Exclusion Criteria	Filters out participants with pre-existing spinal pathologies not related to computer use (e.g., trauma, recent surgery, or non-ergonomic conditions).
Section E	Work Pattern Identification	Identifies the professional's routine as Sedentary, Mobile, or Hybrid with bilingual definitions.
Section F	Stage-specific Symptom Identification	A comprehensive list of Stage 1 and Stage 2 symptoms in English and Hindi, rated on a 0–3 severity scale.
Section G	Participant Declaration	Confirmation of data accuracy for research and academic purposes.
